# Outcome of Percutaneous Endoscopic Lumbar Discectomy in Relation to the Surgeon’s Experience: Propensity Score Matching

**DOI:** 10.3390/bioengineering11040312

**Published:** 2024-03-26

**Authors:** Seong Son, Michael Y. Oh, Han Byeol Park, Alexander M. Lopez

**Affiliations:** 1 Department of Neurosurgery, Gil Medical Center, Gachon University College of Medicine, Incheon 21565, Republic of Korea; sonseong44@gmail.com (S.S.);; 2 Department of Neurological Surgery, University of California, Irvine, CA 92697, USA

**Keywords:** complications, endoscope, learning curve, lumbosacral region, percutaneous discectomy, treatment outcome

## Abstract

Percutaneous endoscopic lumbar discectomy (PELD) presents a challenging learning curve, and the correlation between surgeon experience and clinical outcomes remains contentious. This retrospective study aimed to compare the outcomes of PELD performed by a single surgeon at beginner and experienced stages. Propensity score matching selected 150 patients (75 per group) with a minimum 3-year follow-up. Clinical and radiological outcomes, perioperative complications, and adverse events were assessed. Baseline characteristics, pain improvement, patient satisfaction, and radiological outcomes did not differ between the groups. However, operation time was longer in the beginner group than in the experienced group (57.5 min [IQR, 50.0–70.0] versus 50.0 min [IQR, 45.0–55.0], *p* < 0.001). The beginner group had higher perioperative complication rates (eight patients [10.7%] versus one patient [1.3%], with a hazard ratio of 8.836 [95% CI, 1.077–72.514], *p* = 0.034) and lower 3-year survival without adverse events (19 patients [25.3%] in the beginner group and 10 patients [13.3%] in the experienced group, *p* = 0.045). Our findings indicate that the clinical outcomes were more favorable in patients operated on at the experienced stage compared to those treated at the beginner stage.

## 1. Introduction

Percutaneous endoscopic lumbar discectomy (PELD), including transforaminal approach (transforaminal endoscopic lumbar discectomy (TELD)), interlaminar approach (interlaminar endoscopic lumbar discectomy (IELD)), and foraminal approach (extraforaminal endoscopic lumbar discectomy (EELD)), are one of the most proven minimally invasive surgeries for lumbar disc herniation using water-based uniportal endoscopy [1]. The representative advantages of PELD in comparison with microscopic surgery include small incisions, minimal soft tissue damage, and rapid recovery following surgery [2,3]. Although there is controversy regarding complications and reoperation rates [4,5], the overall clinical outcomes of PELD were found to be similar or superior to those of microscopic surgery [6,7,8]. 

One of the barriers for a beginner surgeon to perform PELD is the difficult learning process [9]. Generally, spine surgeons need to become more familiar with full-endoscopic spine surgery due to the different approaches and trajectories, limited vision through a narrow endoscope, and two-dimensional view through an inclined window [10]. Consequently, the technical demands of PELD, such as the need for the precise landing of the endoscope, visualization of the surgical field, and appropriate instrument control, require a high level of manual dexterity and experience. Based on these concepts, previous studies reported that the required cases and time to acclimate to full-endoscopic techniques are larger than conventional surgery [11,12,13]. 

Although this difficult learning process can affect the outcomes of PELD, controversy exists regarding the clinical outcomes related to surgical proficiency. Several studies suggest that the overall outcomes of PELD were similar despite the difference in operation time between beginner and expert surgeons [14,15,16,17]. Contrarily, some studies have demonstrated that the surgeon’s expertise and experience influence the outcomes of PELD, including surgical success, complication rates, and reoperation rates [18,19]. However, limited previous studies have directly compared PELD outcomes between non-experienced and experienced stages of surgical proficiency, with insufficient scientific evidence available. Prior investigations have been constrained by factors such as surgeon heterogeneity, small sample sizes, short-term follow-up periods of less than 1 year, or selection bias, hampering comprehensive comparative analysis [14,15,16,17,18,19]. Hence, there is a need for methodological studies comparing outcomes based on a single surgeon’s skill level. 

We hypothesized that experts would demonstrate superior results compared to beginners. In this study, we retrospectively compared the outcomes of PELD performed by a single surgeon at two different stages of surgical proficiency, beginner and experienced, with a follow-up period of 3 years. After employing propensity score matching (PSM) to minimize selection bias, we focused on perioperative complications, adverse events, and quantitative outcomes.

## 2. Materials and Methods

### 2.1. Study Design and Ethics

This single-center, retrospective study using PSM was conducted in line with global/local ethics. The study protocols were approved by the Institutional Review Board of our institute (GAIRB2023-188). The requirement for informed consent from patients was waived due to the study’s retrospective design.

### 2.2. Surgical Indication of PELD and Surgeon Profile

The surgical indications of PELD for lumbar disc herniation included the following criteria: confirmed lumbar disc herniation compressing nerve root or thecal sac significantly, as determined by preoperative magnetic resonance imaging (MRI); persistent leg pain despite a minimum of 6 weeks of conservative treatment, which encompassed pain medication, physical therapy, and interventions, such as epidural injections or nerve root blocks; and extreme pain that renders daily activity impossible or severe pain accompanied by at least 3/5 motor weakness regardless of the duration of conservative treatment. 

The PELD procedures were performed by a single surgeon at a single institute. The surgeon began performing PELD in September 2014, following a comprehensive career path that included a 4-year residency, a 2-year fellowship, and 1.5-year experience as an independent clinician specializing in spine surgery. During the fellowship and independent clinician period, the surgeon underwent intermittent mentoring from an expert in full-endoscopic surgery and several intensive cadaveric training courses to enhance skills in PELD techniques. 

### 2.3. Patient Sample and Grouping

A total of 225 patients who underwent TELD, IELD, or EELD for lumbar disc herniation by a single surgeon between September 2014 and August 2019 were screened. The study’s exclusion criteria included prior surgery at the same level, multi-level surgery, insufficient follow-up of at least 3 years, and incomplete medical or imaging records. After applying the exclusion criteria, a final cohort of 187 patients was recruited for the study. 

**Figure 1:**
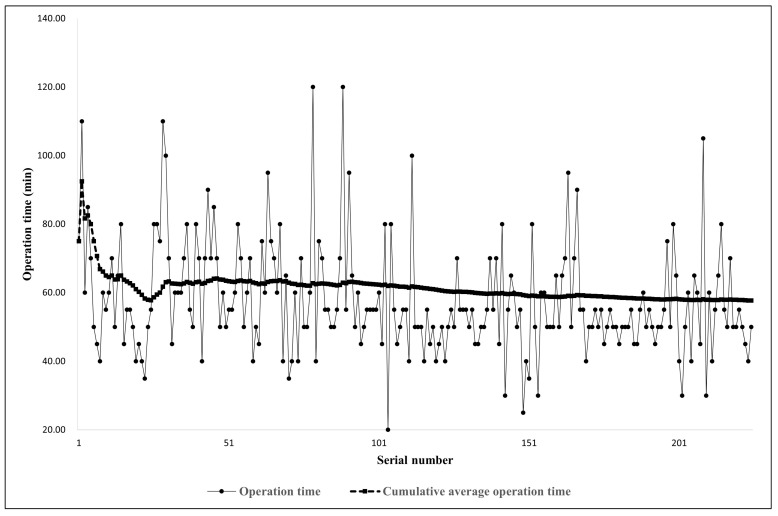
Trends of operation time according to serial number of cases.

Operation time, measured from skin incision to wound closure, was collected for all patients to determine grouping. The median operation time among all patients was 55.00 (interquartile range [IQR], 50.00–65.00) min, and a trend of diminishing operation time was observed with the accumulation of cases. Additionally, the cumulative average operation time demonstrated a plateau, converging to 57.67 min with the accumulation of surgical cases. Furthermore, linear regression analysis revealed a linear functional decrease in operation time as the serial number increased, represented by the following equation: operation time = 63.229 − (0.145 × serial number) (*p* = 0.014, R^2^ = 0.027). In other words, there was a quantitative correlation between surgical proficiency based on operation time and cumulative surgical experience over time (Figure 1). 

Based on these findings, the study cohort was divided into two groups, reflecting the period of the surgeon’s experience and surgical skill level, with a cutoff at the halfway point of 5 years of total experience. Accordingly, 82 patients who underwent PELD during the first 2.5 years of the surgeon’s career (between September 2014 and February 2017) were assigned to the beginner group, while 105 patients who underwent PELD during the second 2.5 years of the surgeon’s career (between March 2017 and August 2019) were assigned to the experienced group. The median operation time was 60.00 (IQR, 50.00–70.00) min in the beginner group and 50.00 (IQR, 50.00–55.00) min in the experienced group (*p* < 0.001, nonparametric Mann–Whitney U test). 

### 2.4. Data Collection

We collected the patients’ baseline characteristics, including age, sex, body mass index, smoking status, alcohol intake, surgery level, and type of procedure (TELD, IELD, or EELD). In addition, we collected baseline data related to symptoms, including symptom sidedness, duration, and motor strength. Furthermore, we surveyed the clinical course before surgery, including previous interventions and trauma affecting symptom aggravation.

The degree of pain was assessed using the visual analog scale (VAS) of back pain/leg pain. The VAS was collected preoperatively and 4 weeks ± 1 week, 1 year ± 1 month, and 3 years ± 3 months following surgery. In addition, patient satisfaction was estimated using Odom’s criteria at each follow-up visit [20]. 

Magnetic resonance imaging (MRI) was performed before and immediately after surgery in all patients to assess nerve decompression or any complications. The degree of preoperative disc degeneration was evaluated using the Pfirrmann grade [21], and the type of ruptured disc was determined as either migrated or subligamentous based on the preoperative MRI. 

Lumbar plain X-rays were performed preoperatively and 3 years ± 3 months following surgery. The mean disc height of the surgery level was calculated as the average of the anterior, middle, and posterior disc heights. To compensate for the variation in X-ray magnification, the disc height ratio to the vertebral body (%) was defined as the ratio of the mean disc height to the anteroposterior diameter of the L5 vertebral body [22]. Additionally, the segmental angle and range of motion at the surgery level and the lumbar lordosis and range of motion of the entire lumbar spine were measured using the Cobb method. 

Regarding surgery-related outcomes, we collected data on the bone work performed during surgery (e.g., foraminoplasty, pediculectomy, or partial endplate resection) and the operation time from skin incision to wound closure. Preoperative hemoglobin levels and postoperative hemoglobin levels 1 day after surgery were collected to indirectly assess blood loss during surgery. We also recorded the patients’ hospital stays to estimate the recovery period. 

We meticulously investigated perioperative complications and adverse events during the 3-year study period. Perioperative complications included surgery-related complications (e.g., exiting root irritation/injury, durotomy, nerve damage, and surgical site infection) and non-surgery-related complications (e.g., cardiopulmonary complications, deep vein thrombosis, and urinary retention). Adverse events during follow-up included surgical failure and conversion to open surgery, a remnant lesion causing persistent symptoms, recurrence in the same lesion site, additional admission for care, additional nerve block for pain control, revision surgery for the same lesion, and revision surgery for another lesion. We defined a remnant lesion if there was further treatment, including reoperation or additional intervention, due to a significant residual nerve compression observed on the postoperative MRI and persistent pain after surgery. 

### 2.5. PSM and Grouping 

To balance the baseline characteristics, PSM analysis was conducted using SPSS version 27.0 (IBM^®^, Armonk, NY, USA). The covariates included age, symptom duration, type of procedure, preoperative hemoglobin level, preoperative Pfirrmann grade, preoperative disc height ratio to the vertebral body, preoperative segmental angle and range of motion at the surgery level, and lumbar lordosis and range of motion of the entire lumbar spine. 

**Figure 2:**
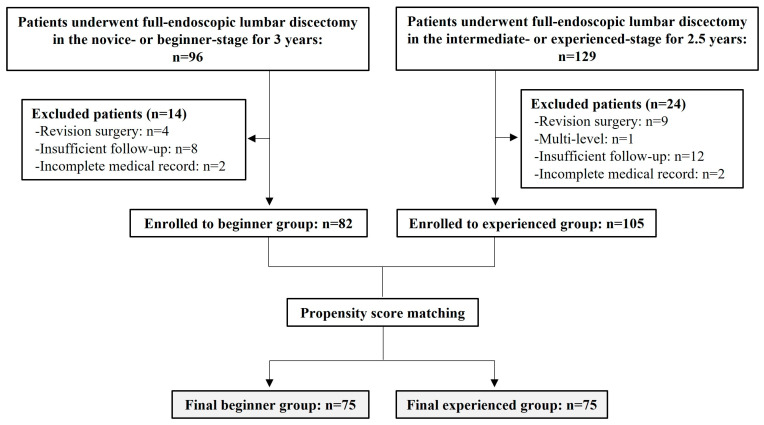
Patient selection process according to propensity score matching. EELD: extraforaminal endoscopic lumbar discectomy; IELD: interlaminar endoscopic lumbar discectomy; PELD: percutaneous endoscopic lumbar discectomy; TELD: transforaminal endoscopic lumbar discectomy.

After PSM, 75 patients were allocated to either the beginner or experienced group (Figure 2). 

### 2.6. Statistical Analysis 

Statistical analyses were conducted using SPSS version 27.0 (IBM^®^, Armonk, NY, USA). We employed independent *t*-test, paired *t*-test, nonparametric Mann–Whitney U test, nonparametric Wilcoxon signed-rank test, Fisher’s exact test, Pearson’s chi-square test, and Kaplan–Meier survival analysis based on the characteristic of the values. The results were expressed as means ± standard deviation, means with a 95% confidence interval (CI), or medians with IQR depending on their distribution. A *p*-value of less than 0.05 was considered statistically significant. 

## 3. Results

### 3.1. Baseline Characteristics 

All baseline characteristics were not significantly different between the two groups (Table 1). 

### 3.2. Pain Improvement and Patient Satisfaction

The VAS score for back pain significantly improved at 4 weeks following surgery in both groups (from a median of 6.5 [IQR, 0.0–8.0] to 2.0 [IQR, 1.0–3.0] in the beginner group and from 6.0 [IQR, 0.0–7.5] to 2.0 [IQR, 0.0–2.75] in the experienced group, *p* < 0.001, nonparametric Wilcoxon signed-rank test). Similarly, the VAS score for leg pain significantly improved at 4 weeks following surgery in both groups (from a median of 7.5 [IQR, 7.0–9.0] to 2.0 [IQR, 1.0–4.0] in the beginner group and from 8.0 [IQR, 7.0–9.0] to 2.0 [IQR, 2.0–3.0] in the experienced group, *p* < 0.001, nonparametric Wilcoxon signed-rank test). However, no intergroup difference was observed in the VAS back and VAS leg in all survey points (Table 2).

As measured by Odom’s criteria, patient satisfaction was favorable in both groups, with 86.67–97.33% of patients reporting excellent or good satisfaction following surgery. However, no intergroup difference was observed in the distribution of patient satisfaction at all survey points (Table 2).

### 3.3. Radiological Outcomes 

Preoperative disc degeneration and the type of ruptured disc were not different between the two groups. The disc height ratio to vertebral body significantly decreased at 3 years following surgery in both groups longitudinally (from a mean of 29.26 ± 10.25% to 28.57 ± 9.91% in the beginner group and from 27.81 ± 7.32% to 27.28 ± 9.06% in the experienced group, *p* < 0.001, paired *t*-test). However, the radiological outcomes based on plain X-rays were not significantly different between the two groups (Table 3). 

### 3.4. Surgery-Related Outcomes

There was no statistically significant difference in bone work during surgery between the two groups, although bone work was more frequent in the experienced group. The operation time was significantly longer in the beginner group than in the experienced group (median 57.5 [IQR, 50.0–70.0] min versus 50.0 [IQR, 45.0–55.0] min, *p* = 0.001, nonparametric Mann–Whitney U test). Although the preoperative hemoglobin level was not different between the two groups, the postoperative hemoglobin level was higher in the beginner group than in the experienced group (mean 14.20 ± 1.57 g/dL versus 13.47 ± 1.56 g/dL, *p* = 0.015, independent *t*-test), and the decrease in hemoglobin level following surgery was smaller in the beginner group than in the experienced group (median 0.30 [IQR, −0.15–1.05] versus 0.80 [IQR, 0.50–1.00], *p* = 0.024, nonparametric Mann–Whitney U test). On the other hand, hospital stays did not significantly differ between the two groups (Table 4). 

### 3.5. Perioperative Complications and Adverse Events

The incidence of perioperative complications was significantly different between the two groups. Eight patients (10.67%) in the beginner group and one [1.33%] in the experienced group underwent complications, with a hazard ratio of 8.836 (95% CI, 1.077–72.514]) (*p* = 0.034, Fisher’s exact test). Surgery-related complications occurred in seven patients (9.33%) in the beginner group (transient exiting root irritation in four, iatrogenic durotomy in two, and surgical site infection in one). In contrast, only one patient (1.33%) in the experienced group had a surgical site infection with discitis (*p* = 0.063, Fisher’s exact test). Regarding non-surgery-related complications, one patient in the beginner group experienced transient bladder distention following surgery (Table 5).

Although not statistically significant, there was a trend toward a larger number of adverse events during the 3-year follow-up period in the beginner group compared with the experienced group (19 patients [25.33%] in the beginner group versus 10 patients [13.33%] in the experienced group, *p* = 0.063, Pearson’s chi-square test). In the beginner group, one conversion to open surgery occurred due to the intraoperative finding of intradural disc rupture. Remnant lesions requiring further treatment occurred in three patients (4.00%) in the beginner group and none in the experienced group (*p* = 0.245, Fisher’s exact test). Recurrent disc herniation with symptom aggravation occurred in six patients (8.00%) in the beginner group and eight patients (10.67%) in the experienced group (*p* = 0.593, Pearson’s chi-square test). Revision surgery for the previously treated lesion was performed in nine patients (12.00%) in the beginner group and eight patients (10.67%) in the experienced group (*p* = 0.797, Pearson’s chi-square test). The same-site revision surgery in the beginner group included four cases of revisional PELD for recurrence, two cases of revisional microscopic discectomy for recurrence, one case of fusion surgery for recurrence, and two cases of microscopic discectomy for a remnant lesion. The experienced group had four cases of revisional PELD for recurrence, two cases of revisional microscopic discectomy for recurrence, one case of fusion surgery for recurrence, and one case of fusion surgery for iatrogenic discitis. In addition, revision surgery for another lesion was performed in one patient (1.33%) with disc herniation at an adjacent cranial level at 3 months following surgery in the beginner group. In contrast, no patient underwent revision surgery for another lesion in the experienced group (*p* = 1.000, Fisher’s exact test) (Table 5). 

**Figure 3:**
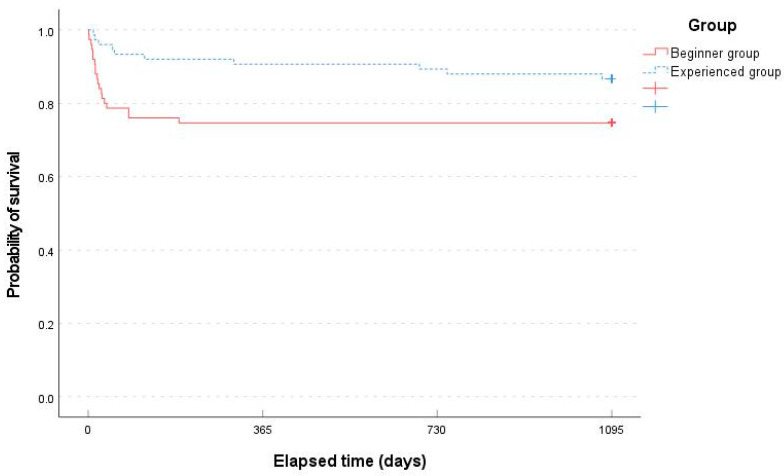
Comparison of the occurrence of adverse events between the two groups over 3 years based on Kaplan–Meier survival analysis. + status at the end of follow-up.

According to the Kaplan–Meier survival analysis of any adverse events during the 3-year follow-up period, the mean time to occurrence of an event was significantly different between the two groups (826.04 days [95% CI, 721.42–930.66] in the beginner group and 990.27 days [95% CI, 922.61–1057.93] in the experienced group, *p* = 0.045, log-rank test) (Figure 3). 

**Figure 4:**
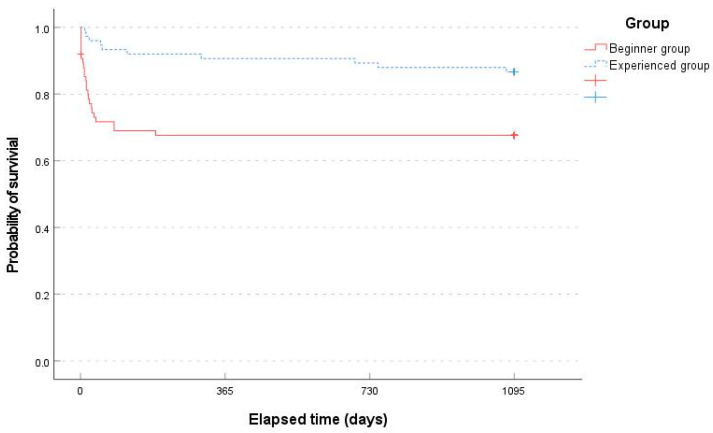
Comparison of perioperative complications or adverse events between the two groups over 3 years based on Kaplan–Meier survival analysis. + status at the end of follow-up.

Significant intergroup difference was observed for the sum of complications or adverse events during follow-up (24 patients [32.00%] in the beginner group versus 10 patients [13.33%] in the experienced group, with a hazard ratio of 3.059 [95% CI, 1.342–6.697], *p* = 0.006, Pearson’s chi-square test). According to the Kaplan–Meier survival analysis of all perioperative complications and adverse events during the 3-year follow-up period, the mean time to occurrence of an event was significantly different between the two groups (749.37 days [95% CI 635.46–863.27] in the beginner group and 990.27 days [95% CI, 922.61–1057.93] in the experienced group, *p* = 0.003, log-rank test) (Figure 4).

## 4. Discussion

According to the present study, the two groups had no significant differences regarding pain improvement, patient satisfaction, and radiological outcomes during the 3-year follow-up. These findings align with previous reports that have indicated similar overall outcomes regardless of surgical proficiency [14,15,16,17]. However, the complication rate and survival without adverse events were better in the experienced group than in the beginner group. These findings also support previous studies that have suggested a correlation between a surgeon’s experience and improved outcomes in terms of surgical success, complication, and the need for reoperation [18,19]. In summary, while the overall quantitative outcomes remained unaffected, it is evident that surgical proficiency can influence the incidence of complications or adverse events. 

Regarding surgery-related outcomes, although the duration of hospital stay did not differ between the two groups, the operation time was significantly longer in the beginner group compared to the experienced group. This finding is in line with previous research suggesting that operation time is an indicator of surgical proficiency level [23]. However, it remains unclear whether this difference in operation time directly impacts the overall outcome. The reasons behind the difference in postoperative hemoglobin levels and hemoglobin decrease following surgery between the two groups are also unknown. Nevertheless, it is possible that the slightly higher amount of bone work performed during the experienced stage contributed to greater blood loss during surgery. 

For beginners of PELD, there are significant challenges that arise from a fundamentally different approach and anatomical perspective compared to conventional surgery. The previous literature has suggested that an appropriate level of experience for full-endoscopic surgery ranges from 20 to 50 cases and reported either no significant difference or slightly higher difficulty compared to microscopic surgery [11,12,24,25]. However, considering various factors, such as the entirely different surgical approach, the narrow and inclined two-dimensional view, unfamiliar instrument manipulation, potential surgical complications (e.g., exiting root irritation/injury, dura tear, nerve injury, incomplete decompression, and early recurrence), and the stressful intraoperative situations that can occur (e.g., uncertain anatomical landmarks, blurred vision due to bleeding, inaccessibility to target lesion), it becomes apparent that full-endoscopic surgery requires a rigorous learning process [13,18]. In this regard, it is necessary to take a more conservative approach when estimating the number of PELD cases required to attain proficiency [13,18]. In this study, the cumulative average operation time for the 225 screened cases did not reach a plateau at a specific midpoint but gradually decreased as the number of cases increased. Moreover, even when comparing the data based on a cut-off of a 2.5-year practice period with 96 cases performed, significant differences were observed in operation time, complication rates, and survival without adverse events during the study period. Therefore, it is reasonable to assume that achieving stable surgical proficiency in PELD requires more than 2.5 years of practice, with at least 96 cases to be performed. 

To overcome the challenging learning curve of PELD, it is crucial to emphasize the role of surgeon training and mentorship in improving surgical proficiency. It is essential for surgeons to receive comprehensive training and specialized education, such as in-depth cadaver workshops, before practical application to effectively adapt to the full-endoscopic system and understand the nuances of endoscopic anatomy [26,27,28]. Research has shown that working under the supervision of experienced surgeons enhances a beginner’s ability to perform PELD and accelerates their learning curve [29]. Through guidance and mentorship, experienced surgeons can assist novice surgeons in navigating the complexities of PELD and improving their surgical skills.

In addition, surgeons need to be aware of their own surgical skill level. It should be considered that beginners are more likely to face unfavorable situations, such as complications or surgical failure, than expert surgeons. Accordingly, the proper patient and the appropriate surgical approach should be selected based on the surgeon’s surgical proficiency [30]. 

This study has several limitations that should be acknowledged. The retrospective design introduces potential errors, such as the heterogeneity of confounding factors, selection bias, or researcher bias. However, we attempted to minimize these errors and balance the baseline characteristics in the comparative analysis by utilizing data from a single surgeon and applying the PSM method. Additionally, it is important to consider that this study compared two different periods in the surgeon’s career, which may have involved changes in instrument systems or operating room environments. However, we could partially mitigate concerns regarding this aspect because the surgeries were performed using the same instrument system and surgical protocol in a single institute. Furthermore, the sample size was not large enough, and the follow-up period was not sufficiently long. 

While this study provides valuable insights by methodologically analyzing the differences in outcomes based on surgical experience in a single surgeon, it is crucial to note that further large-scale studies with a longer follow-up are necessary to validate these findings. 

## 5. Conclusions

The results of this study indicate that the experience of the surgeon is a significant factor in the outcome of PELD, particularly in terms of complications and adverse events. This study may serve as a motivating factor for novice surgeons to engage in self-evaluation and pursue continuous training in full-endoscopic spine surgery. 

## Figures and Tables

**Table 1:** Baseline characteristics.

	Beginner (*n* = 75)	Experienced (*n* = 75)	*p*-Value
Age	46.95 ± 14.70	49.07 ± 15.51	0.392 ^†^
Sex: Male/Female	46/29	39/36	0.249 ^‡^
Height (cm)	168.34 ± 9.58	168.22 ± 9.44	0.930 ^†^
Weight (kg)	69.27 ± 16.21	68.59 ± 12.43	0.750 ^†^
Body mass index (kg/m^2^)	24.28 ± 4.06	24.17 ± 3.54	0.851 ^†^
Smoking: Yes/No	10/65	13/62	0.497 ^‡^
Alcohol: Yes/No	28/47	27/48	0.855 ^‡^
Surgery level: L3-L4/L4-L5/L5-S1	2/56/17	7/45/23	0.087 ^‡^
Type of procedure: TELD/IELD/EELD	66/6/3	62/7/6	0.548 ^‡^
Dominant symptom side: Right/Left/Equivocal	26/48/1	23/52/0	0.654 ^‡^
Symptom duration (weeks)	9.6 (IQR, 2.0–15.7)	13.8 (IQR, 4.3–30.0)	0.228 ^†^
Motor weakness: Yes/No	25/50	21/54	0.776 ^‡^
Previous nerve block: Yes/No	36/39	42/33	0.274 ^‡^
Aggravating trauma: Yes/No	8/67	3/72	0.209 ^‡^

IQR: interquartile range; EELD: extraforaminal endoscopic lumbar discectomy; IELD: interlaminar endoscopic lumbar discectomy; TELD: transforaminal endoscopic lumbar discectomy. ^†^ Independent *t*-test; ^‡^ Pearson’s chi-square test.

**Table 2:** Pain improvement and patient satisfaction during follow-up.

	Beginner (*n* = 75)	Experienced (*n* = 75)	*p*-Value
VAS back			
Preoperative	6.5 (IQR, 0.0–8.0)	6.0 (IQR, 0.0–7.5)	0.770 ^†^
4 weeks	2.0 (IQR, 1.0–3.0)	2.0 (IQR, 0.0–2.75)	0.679 ^†^
1 year	1.0 (IQR, 0.0–1.0)	0.0 (IQR, 0.0–1.0)	0.592 ^†^
3 years	1.0 (IQR, 0.0–1.0)	0.0 (IQR, 0.0–1.75)	0.732 ^†^
VAS leg			
Preoperative	7.5 (IQR, 7.0–9.0)	8.0 (IQR, 7.0–9.0)	0.869 ^†^
4 weeks	2.0 (IQR, 1.0–4.0)	2.0 (IQR, 2.0–3.0)	0.826 ^†^
1 year	1.0 (IQR, 0.0–2.0)	1.0 (IQR, 0.0–2.0)	0.725 ^†^
3 years	0.0 (IQR, 0.0–1.0)	1.0 (IQR, 0.0–2.0)	0.432 ^†^
Odom’s criteria: Excellent/Good/Fair/Poor			
4 weeks with success rate	31/34/10/0 (86.67%)	29/44/2/0 (97.33%)	0.078 ^‡^
1 year with success rate	53/18/4/0 (94.67%)	62/12/1/0 (98.67%)	0.157 ^‡^
3 years with success rate	65/9/1/0 (98.67%)	69/5/1/0 (98.67%)	0.423 ^‡^

VAS: visual analogue scale; IQR: interquartile range. ^†^ Nonparametric Mann–Whitney U test; ^‡^ Pearson’s chi-square test.

**Table 3:** Radiological outcomes during follow-up.

	Beginner (*n* = 75)	Experienced (*n* = 75)	*p*-Value
Pfirrmann grade: III/IV/V	46/26/3	32/41/2	0.213 ^†^
Type of ruptured disc: Migrated/Subligamentous	54/21	56/19	0.875 ^†^
Disc height ratio to vertebral body (%)			
Preoperative	29.26 ± 10.25	27.81 ± 7.32	0.362 ^‡^
3 years	28.57 ± 9.91	27.28 ± 9.06	0.449 ^‡^
Segmental angle of the surgery level (°)			
Preoperative	11.35 ± 6.51	11.61 ± 6.47	0.824 ^‡^
3 years	11.38 ± 5.04	12.06 ± 4.99	0.450 ^‡^
Range of motion of the surgery level (°)			
Preoperative	6.95 ± 6.92	5.55 ± 5.32	0.208 ^‡^
3 years	6.14 ± 6.09	4.38 ± 4.00	0.075 ^‡^
Segmental angle of the lumbar spine (°)			
Preoperative	35.65 ± 11.26	36.06 ± 15.24	0.864 ^‡^
3 years	36.00 ± 11.35	38.48 ± 11.45	0.227 ^‡^
Range of motion of the lumbar spine (°)			
Preoperative	24.50 ± 15.50	22.69 ± 13.26	0.482 ^‡^
3 years	27.85 ± 15.37	24.20 ± 10.66	0.124 ^‡^

^†^ Pearson’s chi-square test; ^‡^ independent *t*-test.

**Table 4:** Surgery-related outcomes.

	Beginner (*n* = 75)	Experienced (*n* = 75)	*p*-Value
Bone work during surgery: Yes/No	14/61	22/53	0.126 ^†^
Operation time	57.5 (IQR, 50.0–70.0)	50.0 (IQR, 45.0–55.0)	0.001 ^‡^
Hemoglobin level (g/dL)			
Preoperative hemoglobin	14.49 ± 1.57	14.15 ± 1.52	0.223 ^⸹^
Postoperative hemoglobin	14.20 ± 1.57	13.47 ± 1.56	0.015 ^⸹^
Decrease in hemoglobin	0.30 (IQR, −0.15–1.05)	0.80 (IQR, 0.50–1.00)	0.024 ^‡^
Hospital stays (days)	3.0 (IQR, 3.0–6.8)	4.0 (IQR, 3.0–5.0)	0.064 ^‡^

IQR: interquartile range. ^†^ Pearson’s chi-square test; ^‡^ nonparametric Mann–Whitney U test; ^⸹^ independent *t*-test.

**Table 5:** Perioperative complications and adverse events during follow-up.

	Beginner (*n* = 75)	Experienced (*n* = 75)	*p*-Value
Perioperative complication			
Surgery-related	7 (9.33%)	1 (1.33%)	0.063 ^†^
Non-surgery-related	1 (1.33%)	0	1.000 ^†^
Number of patients who underwent any complication	8 (10.67%)	1 (1.33%)	0.034 ^†^
Adverse events			
Conversion to open surgery	1 (1.33%)	0	1.000 ^†^
Remnant lesion	3 (4.00%)	0	0.245 ^†^
Recurrence	6 (8.00%)	8 (10.67%)	0.593 ^‡^
Additional admission and conservative treatment	1 (1.33%)	1 (1.33%)	1.000 ^†^
Additional nerve block	7 (9.33%)	1 (1.33%)	0.063 ^†^
Revision surgery of previous lesion	9 (12.00%)	8 (10.67%)	0.797 ^‡^
Revision surgery of another lesion	1 (1.33%)	0	1.000 ^†^
Number of patients who underwent any adverse event	19 (25.33%)	10 (13.33%)	0.063 ^‡^
Sum of patients with any complication or adverse event	24 (32.00%)	10 (13.33%)	0.006 ^‡^

^†^ Fisher’s exact test; ^‡^ Pearson’s chi-square test.

## Data Availability

The data presented in this study are available from the corresponding author on receipt of reasonable request and are not publicly available.
